# Evaluation of Next-Generation H3 Influenza Vaccines in Ferrets Pre-Immune to Historical H3N2 Viruses

**DOI:** 10.3389/fimmu.2021.707339

**Published:** 2021-08-12

**Authors:** James D. Allen, Ted M. Ross

**Affiliations:** ^1^Center for Vaccines and Immunology, University of Georgia, Athens, GA, United States; ^2^Department of Infectious Diseases, University of Georgia, Athens, GA, United States

**Keywords:** influenza, H3N2, hemagglutinin vaccine, COBRA, imprinting, pre-immunity, broadly reactive antibody

## Abstract

Each person has a unique immune history to past influenza virus infections. Exposure to influenza viruses early in life establishes memory B cell populations that influence future immune responses to influenza vaccination. Current influenza vaccines elicit antibodies that are typically strain specific and do not offer broad protection against antigenically drifted influenza strains in all age groups of people. This is particularly true for vaccine antigens of the A(H3N2) influenza virus subtype, where continual antigenic drift necessitates frequent vaccine reformulation. Broadly-reactive influenza virus vaccine antigens offer a solution to combat antigenic drift, but they also need to be equally effective in all populations, regardless of prior influenza virus exposure history. This study examined the role that pre-existing immunity plays on influenza virus vaccination. Ferrets were infected with historical A(H3N2) influenza viruses isolated from either the 1970’s, 1980’s, or 1990’s and then vaccinated with computationally optimized broadly reactive antigens (COBRA) or wild-type (WT) influenza virus like particles (VLPs) expressing hemagglutinin (HA) vaccine antigens to examine the expansion of immune breadth. Vaccines with the H3 COBRA HA antigens had more cross-reactive antibodies following a single vaccination in all three pre-immune regimens than vaccines with WT H3 HA antigens against historical, contemporary, and future drifted A(H3N2) influenza viruses. The H3 COBRA HA vaccines also induced antibodies capable of neutralizing live virus infections against modern drifted A(H3N2) strains at higher titers than the WT H3 HA vaccine comparators.

## Introduction

Influenza viruses induce a major respiratory disease that causes ~3-5 million cases of severe illness and 290,000 – 600,000 deaths globally every year (1–5). Currently, vaccines are the most effective measure at preventing influenza virus infections. However, viral infections in vaccinated individuals are still common with antigenically distinct viral strains and subtypes (1, 6, 7). Most commercial influenza virus vaccines elicit antibodies against the major surface proteins, hemagglutinin (HA) and neuraminidase (NA) (1, 8–10). These surface proteins continuously acquire antigenically relevant HA and NA substitutions through the process of antigenic drift, requiring frequent updates to the seasonal influenza vaccine formulations (1, 11, 12). Recently, this rapid evolution has been most evident in viruses belonging to the A(H3N2) subtype. A(H3N2) influenza viruses were first introduced to the human population in 1968 and since then they have undergone substantial antigenic drift leading to numerous seasonal epidemics (13). In the last 20 years, the A(H3N2) component of the Northern Hemisphere vaccine has been changed 13 times, nearly twice as often the A(H1N1) component (14). This rapid evolution makes annual A(H3N2) vaccine strain selection difficult and can lead to the inclusion of mismatched strains in seasonal influenza virus vaccines (13, 15, 16). In the 2018-2019 influenza season, a mismatch between the selected vaccine strain and circulating A(H3N2) viruses led to a vaccine efficacy of ~9%, compared to ~44% for A(H1N1) (14, 17, 18).

The effectiveness of influenza virus vaccines is known to fluctuate from season to season and from person to person based on age and vaccination history (10, 19, 20). In 1960, Francis introduced the concept of “original antigenic sin” (OAS), to describe patterns of antibody responses following vaccination, whereby antibody responses to influenza viruses encountered early in life are preferentially recalled upon exposure to antigenically distinct influenza viral strains (19, 21–23). OAS is a crucial factor that helps shape the immune responses in an individual within and between subtypes of influenza (21). Most humans are infected with influenza viruses by 3-4 years of age and are either infected and or vaccinated with antigenically distinct viral strains later in life resulting in extensive immune memory to historical influenza viruses (1, 4, 6, 8). Human antibody responses are often biased toward the HA epitopes present on the viral strains that were first encountered in childhood, but as people grow older, antibodies to additional families of viruses are acquired (1, 22). Therefore, birth year can have a considerable impact on a person’s future immune responses against drifted stains of influenza virus. This can result in age-specific susceptibility to modern isolates of A(H3N2) viruses based on a particular immune history (21, 24).

Current inactivated influenza vaccines (IIV) primarily induce strain-specific antibodies, but typically do not provide protection against antigenically drifted and shifted strains of influenza (2, 25, 26). Thus, a cross-protective vaccine that can provide immediate protection against antigenically drifted influenza isolates is urgently needed (26). Universal influenza vaccine candidates are currently being designed to overcome the problems associated with strain-specific seasonal influenza vaccines by eliciting broad immune responses against antigenically diverse viral strains in individuals with complex immune histories (1, 9). Previously, our group has reported on the development a next-generation algorithm for generating computationally optimized broadly reactive antigens (COBRA) (27) that can be used as influenza HA vaccines for H1, H2, H3, and H5 subtypes (27–36). These broadly reactive vaccines elicit antibodies capable of blocking HA-specific receptor binding, inhibiting infection, and limiting virus induced pathogenesis in immunologically naïve mice and ferrets against a range of seasonal and pandemic influenza strains (12, 28, 37, 38).

Since most humans have extensive immune histories induced by previous influenza virus infections, this study explored how previous exposure to historical A(H3N2) influenza isolates impacted the COBRA HA elicited immune responses against modern A(H3N2) isolates. Ferrets were first infected with A(H3N2) viruses isolated from different eras (the 1970’s, 1980’s, or 1990’s) to model the immune histories of individuals primed with viruses from these decades. Then, these pre-immune ferrets were vaccinated with next generation H3 COBRA HA vaccines or WT H3 HA antigens expressed on the surface of VLPs. Collected sera was evaluated for the ability to neutralize drifted and future isolates of A(H3N2) influenza viruses (27). Vaccinations in all pre-immune animals increased the breadth of anti-influenza virus neutralizing antibodies. Following a single vaccination with COBRA HA antigens, the collected sera had HAI and neutralizing antibodies that recognized a broader number of A(H3N2) vaccine strains and drifted influenza virus isolates than WT vaccine antigens; demonstrating that the COBRA methodology would be highly advantageous for vaccine manufacturers who want their products to generate potent cross-reactive antibody responses following a single dose of vaccine. However, differences in the vaccine-induced antibody breadth and titer were observed between groups based on their initial exposure to A(H3N2) virus, indicating that an individual’s immune history plays a large role in their response to vaccination. In general, animals primed with viruses that were more genetically related to the vaccine antigens had more cross-reactive antibody breadth following vaccination compared to unprimed ferrets.

## Materials and Methods

### Vaccine Preparation

Mammalian HEK 293-T cells were transfected with three individual plasmids expressing either the influenza N3 neuraminidase (A/mallard/Alberta/24/2001, H7N3), the HIV p55 Gag sequence, to serve as the outer membrane of the particle, and one of the various influenza A(H3N2) wild-type HA or H3 COBRA HA expressing plasmids in previously described mammalian expression vectors (39). A mismatched N3 neuraminidase was included to enable budding of the VLPs from the host cell membrane, while at the same time eliminating any potential protection offered by N2, NA antibodies that could be observed in assays using H3N2 viruses. A(H3N2) wild-type influenza HA sequences were obtained from the Global Initiative on Sharing Avian Influenza Data (GISAID) EpiFlu database using MDCK passaged sequences which were inserted into the pTR600 expression vector (30). This included the H3 HA sequences for A/Wisconsin/67/2005 (EPI_ISL_115646) MDCKP2, A/Texas/50/2012 (EPI_ISL_170149) MDCKP1. Additionally, the TJ-2 and TJ-5 COBRA H3 HA sequences, which were generated using the next generation COBRA methodology (27) were inserted into the pTR600 expression vector and synthesized as virus like particles (VLP’s). TJ-2 was designed using H3N2 wild-type HA sequences from 2002-2005, and TJ-5 using H3N2 sequences from 2008-2012 (27). Following transfection, cells were allowed to incubate at 37°C, 5% CO_2_ for 72 hours, at which point supernatants from transiently transfected HEK 293-T cells were collected, centrifuged at 2500 rpm to remove cellular debris, and sterile filtered through a 0.22μm pore membrane. Mammalian VLPs were purified and sedimented by ultracentrifugation on a 20% glycerol cushion at 135,000 x g for 4h at 4°C. VLPs were resuspended in sterile phosphate buffered saline (PBS) and total protein concentration was assessed by conventional bicinchoninic acid assay (BCA) (33). Hemagglutination activity of each preparation of VLPs was determined by adding equal volume of 0.75% guinea pig red blood cells (RBCs) in the presence of 20nM Oseltamivir, to a V-bottom 96-well plate and incubating with serially diluted volumes of VLP’s for 60 minutes at room temperature (RT). The highest dilution of VLP with full agglutination of RBCs was considered the endpoint HA titer.

### Determination of HA Content

A high-affinity, 96-well flat bottom ELISA plate (Immulon 4 HBK, Thermo Fisher, Waltham, MA, USA) was coated with 5-10μg of total protein of VLP and serial dilutions of a recombinant H3 antigen (3006_H3_Vc, Protein Sciences, Meriden, CT, USA) in ELISA carbonate buffer (50mM carbonate buffer, pH 9.5), and the plate was incubated overnight at 4°C on a plate rocker. The next morning, coated plates were washed in PBS with 0.05% Tween-20 (PBST), then non-specific epitopes were blocked with 1% bovine serum albumin (BSA) in PBST solution for 1h at room temperature. Then, the buffer was removed and stalk-specific Group 2 human antibody CR8020 (Sanofi Pasteur, Lyon, France) 1mg/mL, was added to the plate in blocking buffer at a working dilution of 1:4000, and incubated for 1 h at 37°C (40). Plates were then washed, and probed with goat-anti-human IgG horseradish-peroxidase-conjugated secondary antibody (1mg/mL) diluted in blocking buffer at a working dilution of 1:4000 (2040-05, Southern Biotech, Birmingham, AL, USA) for 1h at 37°C. Plates were washed, and then freshly prepared o-phenylenediamine dihydrochloride (OPD) (P8287, Sigma-Aldrich, St. Louis, MO, USA) substrate in citrate buffer (P4922, Sigma-Aldrich, St. Louis, MO, USA) was added to wells for 3-5 minutes, followed by the addition of 3M H_2_SO_4_ stopping reagent. Plates were then read at 492 nm absorbance using a microplate reader (Powerwave XS, Biotek, Winooski, VT, USA) and background was subtracted from negative wells. Linear regression standard curve analysis was performed using the known concentrations of recombinant standard antigen to estimate HA content in VLP lots (19).

### Viruses and HA Antigens

Influenza A(H3N2) viruses were obtained through either the Influenza Reagents Resource (IRR), BEI Resources, the Centers for Disease Control (CDC), or provided by Virapur (San Diego, CA, USA). Viruses were passaged once in the same growth conditions as they were received, in either embryonated chicken eggs or semi-confluent Madin-Darby canine kidney (MDCK) cell cultures as per the instructions provided by the WHO (41). Virus lots were titered with 0.75% guinea pig erythrocytes in the presence of 20nM Oseltamivir, and made into aliquots for single-use applications.

The A(H3N2) 1968-2019 historical influenza vaccine strain viral panel for HAI analysis included the 25 following viral strains: A/Hong Kong/8/1968 (HK/68) egg passage 3 (EP3), A/Port Chalmers/1/1973 (PC/73) EP1, A/Texas/1/1977 (Tx/77) EP2, A/Bangkok/1/1979 (Bgk/79) EP1, A/Mississippi/1/1985 (Miss/85) EP2, A/Sichuan/2/1987 (Sich/87) EP1, A/Shandong/9/1993 (Shan/93) EP1, A/Nanchang/933/1995 (Nan/95) EP3, A/Sydney/05/1997 (Syd/97) EP2, A/Panama/2007/1999 (Pan/99) EP4, A/Fujian/411/2002 (Fuj/02) MDCKP1, A/New York/55/2004 (NY/04) EP6, A/Wisconsin/67/2005 (Wisc/05) EP4, A/Brisbane/10/2007 (Bris/07) EP3, A/Uruguay/716/2007 (Uru/07) EP2, A/Perth/16/2009 (Per/09) EP4, A/Victoria/361/2011 (Vic/11) EP4, A/Texas/50/2012 (Tx/12) EP4, A/Switzerland/9715293/2013 (Switz/13) EP4, A/Hong Kong/4801/2014 (HK/14) EP11, and A/Singapore/IFNIMH-16-0019/2016 (Sing/16) EP3, A/Kansas/14/2017 (Kan/17) EP1, A/Switzerland/8060/2017 (Switz/17) EP1, A/South Australia/34/2019 (SA/19) EP1, A/Hong Kong/2671/2019 (HK/19) EP1.

The panel of 13 co-circulating H3N2 viral variants from the period of 2009-2016 included: A/Victoria/210/2009 (Vic/09) MDCKP3, A/Alabama/05/2010 (Ala/10) MDCKP2, A/Hessen/5/2010 (Hess/10) MDCKP3, A/Netherlands/009/2010 (Neth/10) MDCKP2, A/Norway/1330/2010 (Nor/10) MDCKP3, A/Madagascar/0648/2011 (Mad/11) MDCKP2, A/Norway/1186/2011 (Nor/11) MDCKP2, A/Stockholm/18/2011 (Stock/11) MDCKP3 A/Athens/112/2012 (Ath/12) MDCKP2, A/Jordan/30502/2012 (Jord/12) MDCKP2, A/Denmark/96/2013 (Den/13) MDCKP2, A/Hong Kong/12/2014 (HK/12/14) MDCKP2, A/Stockholm/28/2016 (Stock/16) MDCKP2.

When viruses could not be obtained, H3N1-Gag VLPs were synthesized using codon optimized wild-type H3 HA sequences obtained from GISAID. A mismatched N1 neuraminidase, from A/Thailand/1/2004, was included to enable budding of the viruses from the host cell membranes, while at the same time eliminating any potential protection offered by N2, NA antibodies that could be observed in assays using H3N2 viruses. This included the following 9 antigens for the 2016-2019 co-circulating strains panel: A/Fiji/110/2016 (Fiji/2016) EPI_ISL_289834, A/Georgia/12/2018 (Geo/18) EPI_ISL_329856, A/Nevada/37/2016 (Nev/16) EPI_ISL_237356, A/Moscow/41/2017 (Mos/17) EPI_ISL_276910, A/Washington/50/2017 (Wash/17) EPI_ISL_286341, A/Sao Paulo/690385/2018 (SP/18) EPI_ISL_299888, A/Abu Dhabi/240/2018 (Abu/18) EPI_ISL_338293, A/Colombia/0082/2019 (Col/19) EPI_ISL_356289, and A/Louisiana/39/2019 (LA/19) EPI_ISL_398579.

### Selection of Co-Circulating H3N2 Variants (2009–2019)

Selection of the co-circulating variants was based upon multiple sequence alignments that were performed on HA sequences downloaded from the GISAID database that were aligned and clustered using Geneious bioinformatics software (Biomatters, Ltd. Auckland, New Zealand). Sequences downloaded from 2009-2019 were organized based on collection during representative “Northern” or “Southern” Hemisphere influenza “seasons”. In this approach, the downloaded sequences were separated into periods of time representing the “Northern” and “Southern” Hemisphere influenza seasons spanning from 2009-2019. The “Southern Hemisphere” season was defined to include sequences from all over the globe isolated from 5/1/XX – 9/30/XX of a given year, and each “Northern Hemisphere” season was defined to include sequences from around the world that were isolated from 10/1/XX - 4/30/XY of the following year. For example, sequences included in the 2009 “Southern Hemisphere” collection timeframe included those isolated between 5/1/2009 – 9/30/2009, and the isolates included in the 2009-2010 “Northern Hemisphere” collection contains sequences that were isolated between 10/1/2009 – 4/30/2010, regardless of where the sequences were isolated geographically. Phylogenetic tree models were assembled using a Jukes-Cantor genetic distance model, and a neighbor-joining tree building method in Geneious bioinformatics software. Branches were compared for sequence similarity to the panel of 22 co-circulating viruses. The number of sequences that branched within 95% HA sequence identity of one of the antigens was counted as a cluster, and frequencies of these clusters in each “influenza season” were calculated based on the total number of sequences available per season as described previously (**Figure 5**) (28).

### Viral Infection and COBRA VLP Vaccination of Ferrets

**Figure 1:**
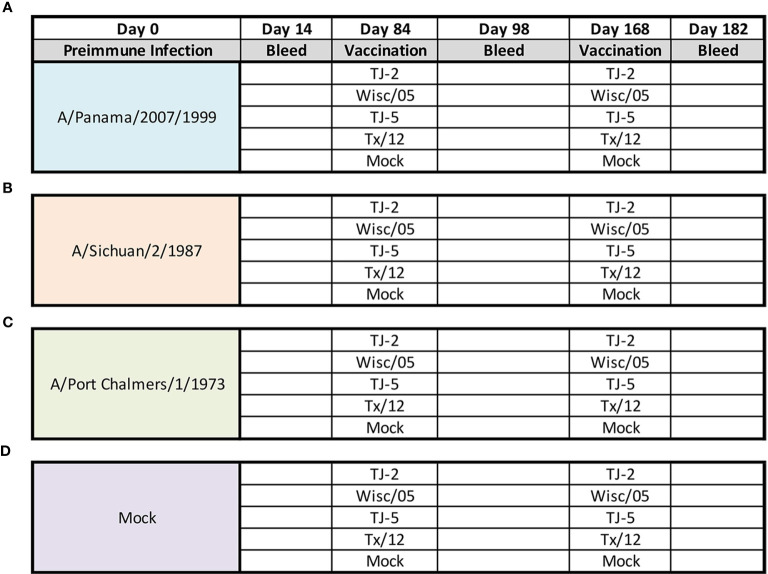
Experimental Design. Eerrets were infected intranasally at day 0 with 1x106 PFU/mL of influenza A(H3N2) virus: A/Panama/2007/1999 **(A)**, A/Sichuan/2/1987 **(B)**, A/Port Chalmers/1/1973 **(C)**, or 1mL of PBS (Mock) **(D)**. At 14 days post infection blood was collected from all animals. After 84 days animals were divided into groups (n=4 ferrets/group) and vaccinated intramuscularly with 15ug of either TJ-2, TJ-5, Tx/12, or Wisc/05 H3N3 VLP’s, or PBS (Mock) mixed 1:1 with adjuvant. At 98 days post infection blood was collected from all animals. At day 168 all animals were boosted with a homologous vaccine to that administered on day 84. A final blood draw was collected at day 182 post infection.

**Figure 2:**
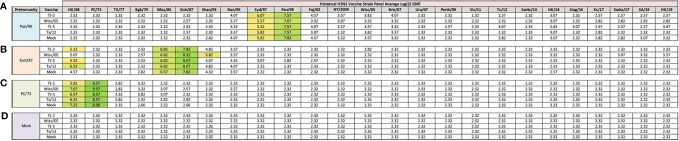
Day 13 H3N2 Historical Vaccine Strain HAI Panel. Serum collected from animals 14 days post infection was analyzed for HAI activity against a panel of historical H3N2 influenza vaccine strains spanning 1968-2019. Table is divided into sections based on the virus each animal received to establish preimmunity: A/Panama/2007/1999 **(A)**, A/Sichuan/2/1987 **(B)**, A/Port Chalmers/1/1973 **(C)**, or PBS (Mock) **(D)**. Cells in table are color coded as a heat map based upon Log(2) HAI geometric mean titer (GMT) for each group of ferrets (N=4). The heat map colors cells yellow at a Log(2) GMT of 5.32 which correspond to an HAI titer of 1:40, and cells become a darker shade of green as the average antibody titer of the group increases. Cells with no correspond to groups that did not achieve a GMT ≥5.32.

Fitch ferrets (*Mustela putorius furo*, female, 6 to 8 months of age), negative for antibodies to circulating influenza A (H1N1, H3N2) and influenza B viruses, were de-scented and purchased from Triple F Farms (Sayre, PA, USA). Ferrets were pair housed in stainless steel cages (Shor-line, Kansas City, KS, USA) containing Sani-Chips laboratory animal bedding (P.J. Murphy Forest Products, Montville, NJ, USA). Ferrets were provided with Teklad Global Ferret Diet (Harlan Teklad, Madison, WI, USA) and fresh water *ad libitum*. Ferrets (n = 4/group) were pre-infected with one of three seasonal H3N2 influenza viruses (1 × 10 (6) PFU) intranasally with 1mL of inoculum diluted in phosphate-buffered saline (PBS, Corning, Tewksbury, MA, USA). One group of ferrets (N=20) was infected with seasonal H3N2 isolate A/Panama/2007/1999 (EP1), another (N=20) with A/Sichuan/2/1987 (EP1) H3N2, and a third (N=20) with A/Port Chalmers/1/1973 (EP1) H3N2 virus. A fourth group (N=20) was mock infected with 1mL of PBS (**Figure 1**). Animals were monitored daily during the infection for adverse events, including weight loss, labored breathing, loss of activity, nasal discharge, sneezing, and diarrhea and allowed to recover for 84 days. Ferrets did not lose more than 5% body weight from any of the pre-infections and serum collected 14 days after the infection was tested in the HAI assay to confirm that animals had seroconverted to the strain used for infection (**Figure 2**). 84 days after the pre-infection, ferrets were vaccinated with 15ug of either one of 2 H3N3 COBRA VLP vaccines (TJ2 or TJ5), one of the wild-type Wisc/05 or Tx/12 H3N3 VLP vaccines, or phosphate-buffered saline alone as a mock vaccination. All the vaccines, (TJ-2, TJ-5, Tx/12, Wisc/05, Mock), were formulated with an emulsified squalene-based oil-in-water emulsion adjuvant, Addavax (InvivoGen, San Diego, CA, USA). The final concentration after mixing 1:1 with VLPs is 2.5% squalene. Ferrets were boosted with an additional 15ug of homologous VLP 84 days after initial vaccination (Day 168). Blood was harvested from all anesthetized ferrets *via* the anterior vena cava at days 14, 84, 98, 168, and 182. Serum was transferred to a centrifuge tube and centrifuged at 2500 rpm for 10 minutes, to separate the serum from the whole blood. Clarified serum was removed and frozen at −20 ± 5°C.

### Hemagglutination-Inhibition (HAI) Assay

The hemagglutination inhibition (HAI) assay was used to assess functional antibodies to the HA that are able to inhibit agglutination of guinea pig erythrocytes. The protocols were adapted from the WHO laboratory influenza surveillance manual (41). Guinea pig red blood cells are frequently used to characterize contemporary A(H3N2) influenza strains that have developed a preferential binding to alpha (2,6) linked sialic acid receptors (42, 43). To inactivate nonspecific inhibitors, sera samples were treated with receptor-destroying enzyme (RDE) (Denka Seiken, Co., Japan) prior to being tested. Briefly, three parts of RDE was added to one part of sera and incubated overnight at 37°C. RDE was inactivated by incubation at 56°C for 30 min. RDE-treated sera were diluted in a series of two-fold serial dilutions in v-bottom microtiter plates. An equal volume of each A(H3N2) virus, adjusted to approximately 8 hemagglutination units (HAU)/50μl in the presence of 20nM Oseltamivir carboxylate, was added to each well. The plates were covered and incubated at room temperature for 30 min, and then 0.75% guinea pig erythrocytes (Lampire Biologicals, Pipersville, PA, USA) in PBS were added. Red blood cells were washed with PBS, stored at 4°C, and used within 24 h of preparation. The plates were mixed by gentle agitation, covered, and the RBCs were allowed to settle for 1 h at room temperature. The HAI titer was determined by the reciprocal dilution of the last well that contained non-agglutinated RBCs. Positive and negative serum controls were included for each plate. All ferrets were negative (HAI ≤ 1:10) for pre-existing antibodies to human influenza viruses prior to vaccination, and for this study sero-protection was defined as HAI titer >1:40 and seroconversion as a 4-fold increase in titer compared to baseline, as per the WHO and European Committee for Medicinal Products to evaluate influenza vaccines (44). The ferrets were naïve and seronegative at the time of vaccination, and thus seroconversion and sero-protection rates are interchangeable in this study.

### Focus Reduction Assay (FRA)

The Focus Reduction Assay (FRA) used in this study was initially developed by the WHO collaborating Centre in London, U.K. and modified by U.S. Centers for Disease Control and Prevention (CDC) (Thomas Rowe, personal communication). MDCK-SIAT1 cells (Sigma, St. Louis, MO, USA) were plated at 2.5 – 3 x 105 cells/ml (100uL/well in 96-well plate) one day prior to use in the assay. Cells were cultured in Dulbecco’s Modified Eagle Medium (DMEM) containing 5% heat-inactivated fetal bovine serum and antibiotics in 96-well flat bottom plates overnight to form a 95-100% confluent monolayer. The following day, the cell monolayers are rinsed with 0.01M phosphate-buffered saline pH 7.2 (PBS, Gibco, Waltham, MA, USA), followed by the addition of 2-fold serially diluted RDE treated serum (50uL per well) starting with a 1:20 dilution in virus growth medium containing TPCK-treated trypsin (1µg/ml), VGM-T, (DMEM containing 0.1% BSA, 1% Penicillin/Streptomycin (100 U/mL Penicillin, 100 ug/mL Streptomycin solution), and 1µg/ml TPCK-treated trypsin) (Sigma, St. Louis, MO, USA). 50uL of A(H3N2) influenza virus (1.2 x 104 focus forming units (FFU)/mL, which corresponds to 600 FFU/50μl) in VGM-T was added to the wells of each plate, or VGM-T only was added to cell control wells. Virus stocks were standardized by previous titration in the FRA (45, 46). Following a 2 h incubation period at 37**°**C with 5% CO**_2_**, the cells in each well were then overlaid with 100uL of equal volumes of 1.2% Avicel RC/CL (Type: RC581 NF; FMC Health and Nutrition, Philadelphia, PA, USA) in 2X Modified Eagle Medium containing 1µg/ml TPCK-treated trypsin, 0.1% BSA and antibiotics (47). Plates were incubated for 18-22 h at 37**°**C, 5% CO_2_. The overlays were then removed from each well and the monolayer was washed once with PBS to remove any residual Avicel. The plates were then fixed with ice-cold 4% formalin in PBS for 30 min at 4**°**C, followed by a PBS wash and permeabilization using 0.5% Triton-X-100 in PBS/glycine at room temperature for 20 min. Plates were washed three times with wash buffer (PBS, 0.1% Tween-20; PBST) and then incubated for 1 h with a monoclonal antibody against the influenza A nucleoprotein (46, 48, 49) obtained from the Influenza Reagent Resource (IRR) (Manassas, VA, USA) (FR-1217) (1mg/mL), diluted 1:2000 in ELISA buffer (PBS,10% horse serum, 0.1% Tween-80). Following washing (3X PBST), the cells were incubated with goat anti-mouse peroxidase-labelled IgG (Sera Care, Inc., Milford, MA, USA) (KPL 474-1802) (1mg/mL), diluted 1:2000 in ELISA buffer for 1 hour at RT. Plates were washed again (3X PBST) and infectious foci were visualized using TrueBlue substrate (Sera Care, Inc., Milford, MA USA) containing 0.03% H**_2_**O**_2_** incubated at room temperature (RT) for 10 min. The reaction was stopped by washing five times with dH_2_0. Plates were air dried and foci enumerated using a CTL BioSpot Analyser with ImmunoCapture 6.4.87 software (CTL, Shaker Heights, OH, USA). The FRA titer was reported as the reciprocal of the highest dilution of serum corresponding to 50% foci reduction compared to the virus control minus the cell control.

In order for a plate to pass quality control, both the average of the octuplet virus control wells (VC), as well as the average of the octuplet cell control wells (CC) must pass. The virus controls initially were between 150 to 650 foci and the cell controls must be free of foci. The virus control wells were subsequently expanded to between 200 and 1600 foci. Additionally, the positive control, A(H3N2) historical influenza vaccine strain, virus was run in triplicate plates in each individual assay and at least two out of three plates must pass VC and CC criteria and homologous ferret antisera, previously generated through infection with A(H3N2) influenza virus at 1e6 FFU/mL and collected 14 days post infection, must have the same titer across the plates (28). Each assay plate (one virus per plate) contained a panel of ferret reference antisera, as well as a human influenza vaccine serum control to assess overall assay consistency (33). The percentage of infected cells reported in the assay is calculated by averaging the foci count from the positive control (virus and cell only) wells, and dividing the number of foci in each experimental well by the average of the positive control.

## Results

### Historical HAI Landscape of VLP Vaccinated Pre-Immune Ferrets

Previously, nine distinct H3 COBRA antigens, TJ-1 – TJ-9 were designed and tested in immunologically naïve mice to determine the breadth of antibody reactivity induced by these universal vaccine candidates against both historical and co-circulating H3N2 influenza strains (27). The results of this investigation led to the selection of two lead candidate COBRA HA immunogens, TJ-2 (designed from WT H3N2 sequences in circulation during 2002-2008) and TJ-5 (designed from WT H3N2 sequences in circulation during 2008-2012), that needed to be tested further in the ferret, a more clinically relevant animal model, to assess their performance in populations with prior influenza immune history. In this study the two next-generation H3 COBRA HA vaccine antigens were compared to two WT historical H3 vaccine antigens, Wisc/05 for comparison to TJ-2, and Tx/12 for comparison to TJ-5, in order to assess their abilities to elicit cross-reactive antibodies in ferrets that are pre-immune to H3N2 viruses representing different historical eras. Ferrets (n=20/group) were infected with a representative virus isolated from the 1973, 1987, or 1999 (**Figure 1**). Sera collected from ferrets at 14 days post-infection had HAI activity against the infecting strain, as well as a few other antigenically related viruses (**Figure 2**). Ferrets infected with the 1990’s representative virus, Pan/99, had antibodies with HAI activity against both Syd/97 and Pan/99. The ferrets infected with the 1980’s representative virus, Sich/87, had antibodies with HAI activity against Miss/85 and Sich/87 with a few serum samples recognizing the HK68 virus and Shan/93. Ferrets infected with the 1970’s representative virus, PC/73, had sera with HAI activity against PC/73 and HK/68. The mock infected influenza naïve ferrets had no HAI activity against any of the 25 H3N2 influenza viruses in the panel (**Figure 2**). Overall, the viruses used to establish pre-immunity elicited the highest antibody titers against the homologous infecting strain with some reactivity to 1 or 2 other viruses in the panel.

**Figure 3:**
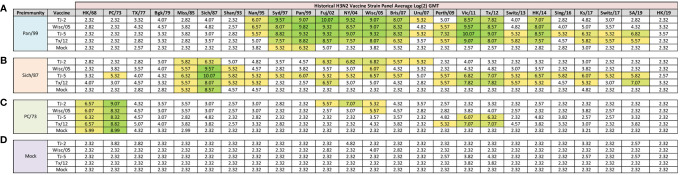
Day 98 H3N2 Historical Vaccine Strain HAI Panel. Serum collected from animals 98 days post infection (14 days after first vaccination) was analyzed for HAI activity against a panel of historical H3N2 influenza vaccine strains spanning 1968-2019. Table is divided into sections based on the virus each animal received to establish preimmunity: A/Panama/2007/1999 **(A)**, A/Sichuan/2/1987 **(B)**, A/Port Chalmers/1/1973 **(C)**, or PBS (Mock) **(D)**. Cells in table are color coded as a heat map based upon Log(2) HAI geometric mean titer (GMT) for each group of ferrets (N=4). The heat map colors cells yellow at Log(2) GMT of 5.32 which correspond to an HAI titer of 1:40 and cells become a darker shade of green as the average antibody titer of the group increases. Cells with no color correspond to groups that did not achieve a GMT ≥5.32.

At 84 days post infection, ferrets were divided into groups (4 animals/group) and then were vaccinated with VLPs expressing either TJ-2, TJ-5, Tx/12, or Wisc/05 H3 HA mixed with Addavax oil-in-water emulsion adjuvant. A mock group was also vaccinated with phosphate buffered saline (PBS) mixed with adjuvant. At day 98 post infection (14 days following vaccination), antisera were collected from the animals and analyzed for HAI activity against a panel of 25 historical H3N2 vaccine strain isolates from 1968-2019 (**Figure 3**). Ferrets pre-immune to Pan/99 and vaccinated with VLPs expressing TJ-2 HA had antibodies with HAI activity against 11/25 (44%) of the strains in the panel including all of the strains from 1995-2007, 2011, 2012, and 2014 (**Figure 3A**). Ferrets pre-immune to Pan/99 and vaccinated with VLPs expressing TJ-5 HA had HAI activity to 17/25 (68%) of the strains comprising all of the isolates from 1995-2019 with the exception of HK/19 (**Figure 3A**). The animals pre-immunized with Pan/99 and vaccinated with VLPs expressing Tx/12 HA HAI activity to 14/25 (56%) of strains comprising strains from 1997-2019, excluding Uru/07, Sing/16, and HK/19 (**Figure 3A**). Similar to the animals vaccinated with VLPs expressing TJ-2 HA, the ferrets pre-immune to Pan/99 and vaccinated with VLPs expressing Wisc/05 HA had HAI against 11/25 (44%) of the strains, including all of the strains from 1995-2014 excluding Uru/07 and Switz/13 (**Figure 3A**). The animals infected with Pan/99 and given a mock vaccination seroconverted to the same strains induced by the infection at day 14, Syd/97 and Pan/99, albeit at slightly lower titers than at day 14 post infection (**Figure 3A**).

Animals pre-immunized with Sich/87 and vaccinated with VLPs expressing TJ-2 HA had antibodies against 7/25 (28%) of the strains. These animals retained the antibodies from the infection with HAI activity against Miss/85 and Sich/87 that were present at day 14, while adding HAI activity against viruses isolated from 2002-2007 (**Figure 3B**). The ferrets infected with Sich/87 and vaccinated with VLPs expressing TJ-5 HA had antibodies with the broadest HAI activity compared to any vaccinated group at day 98, and recognized 20/25 (80%) of the viruses in the panel. These animals seroconverted to PC/73 and all of the strains from 1985-2019 excluding Uru/07 and HK/19 (**Figure 3B**). Ferrets pre-immune to Sich/87 and vaccinated with VLPs expressing Tx/12 HA had HAI activity against 12/25 (48%) of the strains in the panel, including isolates from 1985-1995, Fuj/02, and the strains from isolated between 2009-2019, excluding Sing/16 and HK/19 (**Figure 3B**). Ferrets pre-immune to Sich/87 and vaccinated with VLPs expressing Wisc/05 HA had HAI activity against only 4/25 (16%) of the strains isolated between 1985-1993 and the homologously matched Wisc/05 strain (**Figure 3B**). Ferrets that were only infected with Sich/87 and then mock vaccinated retained their antibody titers to Miss/85 and Sich/87, but did not seroconvert to any of the other strains in the panel (**Figure 3B**). Overall, VLPs with COBRA H3 HA antigens elicited antibodies that recognized more H3N2 influenza strains than VLPs expressing WT H3 HA proteins in the animals that were primed with the Sich/87 virus.

The ferrets infected with PC/73 and vaccinated with VLPs expressing TJ-2 HA had HAI activity against 5/25 (20%) of the H3N2 strains, the two strains from the initial infection, HK/68 and PC/73, as well as the strains from 2002-2005 (**Figure 3C**). Ferrets pre-immune to PC/73 and vaccinated with VLPs expressing TJ-5 HA antisera with HAI activity against 4/25 (16%) of the strains, including the strains from 1968-1973 and 2011-2012 (**Figure 3C**). Animals pre-immune to PC/73 and vaccinated with VLPs expressing WT Tx/12 HA had HAI activity against 5/25 (20%) of the strains, the strains from 1968-1973 and 2009-2012 (**Figure 3C**). Animals pre-immune to PC/73 and vaccinated with VLPs expressing Wisc/05 HA had HAI activity against 3/25 (12%) of the strains in the panel, consisting of viruses isolated from 1968-1973 and the homologously matched Wisc/05 strain (**Figure 3C**). Ferrets that were pre-immunized with PC/73 and administered a mock vaccination retained their antibody HAI activity against HK/68 and PC/73, but did not seroconvert to any of the other strains in the panel (**Figure 3C**). In general, the animals primed with PC/73 had antisera with the least cross-reactive HAI breadth of the three pre-immune regimens. No animals that were mock infected had any HAI seroconversion against any of the strains in the panel following a single VLP vaccination, regardless of the vaccine antigen (**Figure 3D**). The mock pre-immune animals vaccinated with Wisc/05 and Tx/12 do have HAI titers against the homologously matched virus, however these titers are not high enough to yield an average HAI titer of 1:40 or higher for the group (log2 GMT of 4.07 and 3.82 respectively). This is not overly surprising as our group has published on this phenomenon before, where in influenza naïve ferrets more than one vaccination is often required to induce seroconversion to the homologously matched virus (28).

**Figure 4:**
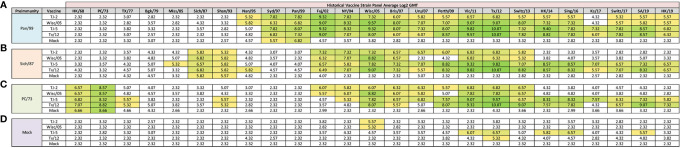
Day 198 H3N2 Historical Vaccine Strain HAI Panel. Serum collected from animals 182 days post infection (14 days after second vaccination) was analyzed for HAI activity against a panel of historical H3N2 influenza vaccine strains spanning 1968-2019. Table is divided into sections based on the virus each animal received to establish preimmunity: A/Panama/2007/1999 **(A)**, A/Sichuan/2/1987 **(B)**, A/Port Chalmers/1/1973 **(C)**, or PBS (Mock) **(D)**. Cells in table are color coded as a heat map based upon Log(2) HAI geometric mean titer (GMT) for each group of ferrets (N=4). The heat map colors cells yellow at Log(2) GMT of 5.32 which correspond to an HAI titer of 1:40 and cells become a darker shade of green as the average antibody titer of the group increases. Cells with no color correspond to groups that did not achieve a GMT ≥5.32.

In order to determine if the cross-reactive breadth elicited by the COBRA vaccine antigens could increase the sero-conversion against more historical H3N2 isolates through repeated vaccination, all ferrets were boosted with a second dose of vaccine at 168 days post infection, and antisera was collected 14 days later, at day 182 (**Figure 4**). Ferrets pre-immune to Pan/99 and vaccinated twice with VLPs expressing TJ-2 HA had HAI activity to 17/25 (68%) of the strains in the panel including of all the strains from 1995-2019 with the exception of Ks/17 (**Figure 4A**). The animals pre-immune to Pan/99 and vaccinated two times with VLPs expressing TJ-5 HA had HAI activity against 17/25 (68%) of the strains, including all of the strains isolated from 1997-2019 (**Figure 4A**). Ferrets pre-immunized with Pan/99 and boosted with VLPs expressing Tx/12 HA had HAI activity against 17/25 (68%) of the H3N2 strains, recognizing the same isolates (1997-2019) as ferrets vaccinated with VLPs expressing TJ-5 HA (**Figure 4A**). Animals pre-immune to Pan/99 and boosted with VLPs expressing Wisc/05 HA seroconverted to 17/25 (68%) of the strains, including all the strains isolated from 1995-2019 with the exception of Ks/17 (**Figure 4A**). The ferrets that were infected with Pan/99 and boosted with mock vaccines retained their HAI activity against Syd/97 and Pan/99, but did not seroconvert against any other strains in the panel (**Figure 4A**).

Animals pre-immunized with Sich/87 and boosted with VLPs expressing TJ-2 HA had antisera with HAI activity against 11/25 (44%) of the strains in the panel, including Sich/87, Shan/93, and all of the isolates from 2002-2013 (**Figure 4B**). The ferrets pre-immune to Sich/87 and boosted with VLPs expressing TJ-5 HA had antisera with HAI activity against 18/25 (72%) of the strains, including all the strains isolated from 1985-1993 and 2002-2019 (**Figure 4B**). Ferrets infected with Sich/87 and boosted with VLPs expressing Tx/12 HA also had antisera with HAI activity against 18/25 (72%) of the strains in the panel, including viruses isolated from 1987-1995 and 2002-2019 (**Figure 4B**). Animals pre-immune to Sich/87 and boosted with VLPs expressing Wisc/05 HA had antisera with HAI activity against 9/25 (36%) of the strains in the panel including isolates from 1987-1993, 2002-2007, and 2011-2013 (**Figure 4B**). Ferrets that were only infected with Sich/87 and given two mock vaccinations retained their antibody HAI activity against Sich/87 and gained HAI activity against Shan/93, but did not seroconvert to any of the other strains in the panel (**Figure 4B**).

Ferrets infected with PC/73 and boosted with VLPs expressing TJ-2 HA had HAI activity against 11/25 (44%) of the H3N2 strains, including HK/68 and PC/73, as well as the isolates from 2002-2013 (**Figure 4C**). The animals pre-immune to PC/73 and boosted with VLPs expressing TJ-5 HA had HAI activity against 18/25 (72%) of the strains in the panel including the viruses isolated between 1968-1977, Shan/93, and all of the isolates from 2004-2019 (**Figure 4C**). Ferrets infected with PC/73 and then boosted with VLPs expressing Tx/12 HA had HAI activity against 15/25 (60%) of the viruses including strains isolated from 1968-1977, Shan/93, and all of the viruses from 2005-2019, excluding Uru/07 and Ks/17 (**Figure 4C**). Ferrets pre-immune to PC/73 were boosted with VLPs expressing Wisc/05 HA had HAI activity against 10/25 (40%) of the strains, including the viruses isolated from 1968-1973 and all of the strains from 2002-2013, excluding Perth/09 (**Figure 4C**). Ferrets that were pre-immunized with PC/73 and mock vaccination retained their HAI activity against HK/68 and PC/73, but had no HA activity against any other strains. in the panel (**Figure 4C**).

Mock infected/naïve ferrets that were vaccinated twice with TJ-5 HA had HAI activity against 5/25 (20%) strains in the panel, including the isolates from 2011-2016, excluding Switz/13 and the SA/19 virus (**Figure 4D**). All mock infected/naïve ferrets that were vaccinated with VLPs expressing either TJ-2, Tx/12, or Wisc/05 HA had HAI activity against only 1/25 (4%) of the strains in the panel, either Tx/12 or Wisc/05 virus (**Figure 4D**). As expected, the mock infected and mock vaccinated ferrets had no HAI activity against any of the strains in the panel (**Figure 4D**). In general, the second vaccination increased the cross-reactive antibody HAI breadth for all of the pre-immune animals, while the naïve animals had limited HAI activity against the panel after the second vaccination.

### Co-Circulating Strain (2009-2019) HAI Landscape of VLP Vaccinated Pre-Immune Ferrets

**Figure 5:**
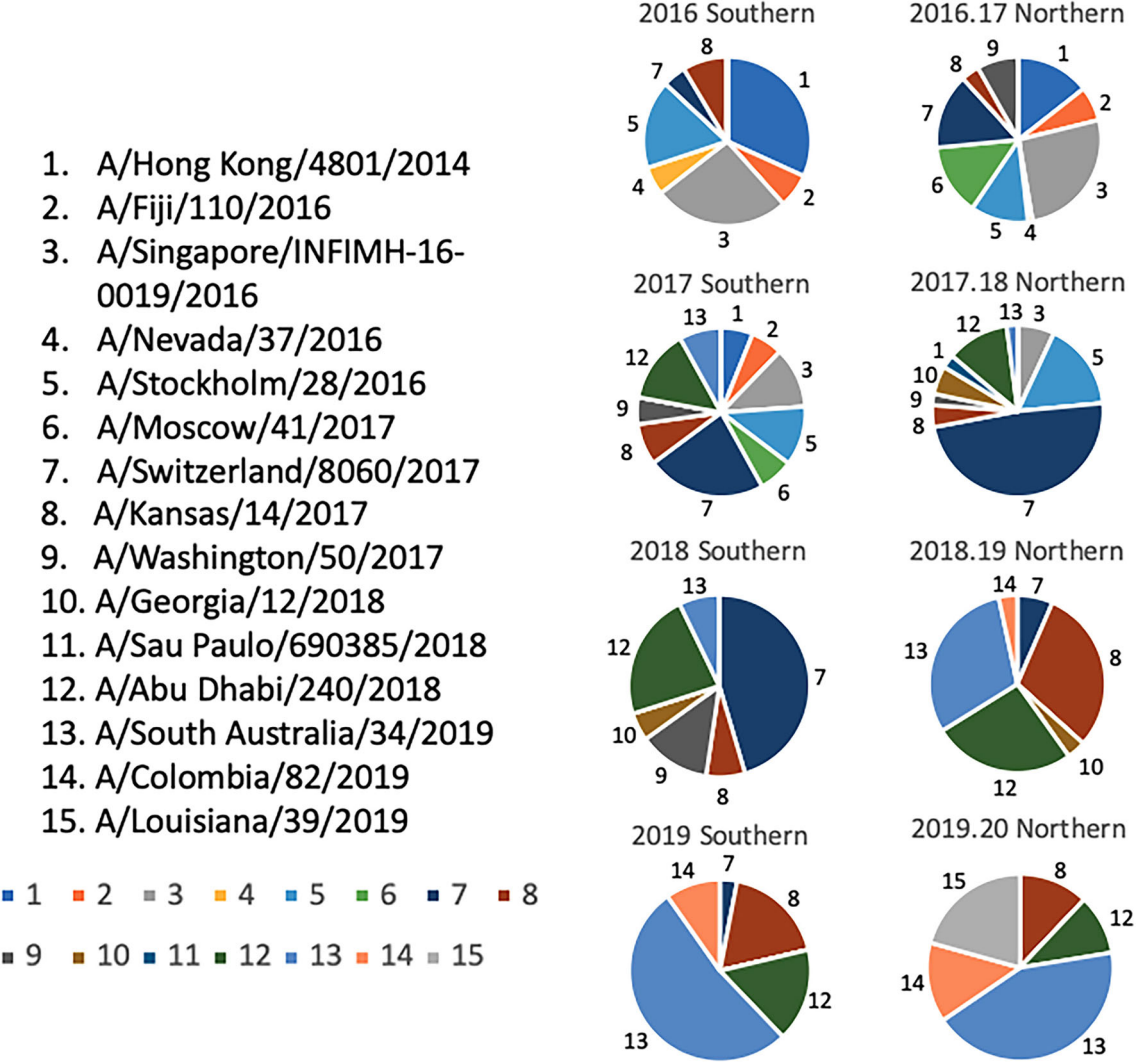
Determination of Co-circulating H3N2 Strains 2016-2020. Relative frequencies of influenza HA clusters from co-circulating H3N2 strains in consecutive seasons from 2016-2020. Influenza HA sequences were obtained from the GISAID database, aligned, and clustered into families for each representative Northern and Southern Hemisphere influenza season timeframe. The HA sequences from each season were then divided into clusters based on a 95% sequence similarity cutoff, and are depicted by a different color and slice of each pie chart. A representative strain from each cluster was chosen and assigned a number 1-15. Colors assigned to each number match the color in the pie charts.

**Figure 6:**
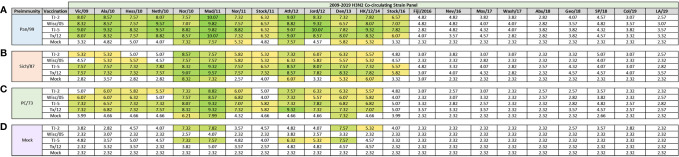
Day 98 H3N2 Co-Circulating Strain HAI Panel. Serum collected from animals 98 days post infection (14 days after first vaccination) was analyzed for HAI activity against a panel of co-circulating H3N2 influenza strains spanning 2009-2019. Table is divided into sections based on the virus each animal received to establish preimmunity: A/Panama/2007/1999 **(A)**, A/Sichuan/2/1987 **(B)**, A/Port Chalmers/1/1973 **(C)**, or PBS (Mock) **(D)**. Cells in table are color coded as a heat map based upon Log(2) HAI geometric mean titer (GMT) for each group of ferrets (N=4). The heat map colors cells yellow at Log(2) GMT of 5.32 which correspond to an HAI titer of 1:40 and cells become a darker shade of green as the average antibody titer of the group increases. Cells with no color correspond to groups that did not achieve a GMT ≥5.32.

Antisera collected from vaccinated ferrets 98 days post-infection was also analyzed in HAI assays across a panel of 15 co-circulating H3N2 influenza drift variants isolated from 2016-2019, as well as 7 H3 drift variants from 2009-2016 which were previously identified (28) (**Figure 5**). Ferrets pre-immune to Pan/99 and vaccinated with VLPs expressing either TJ-2, TJ-5, Tx/12, or Wisc/05 HA proteins all had HAI activity against 13/22 (59.09%) of the co-circulating strains (**Figure 6A**). All ferrets had HAI activity against strains isolated from 2009-2016 excluding Fiji/16, and Nev/16, regardless of which HA vaccine was used for vaccination. None of the vaccines elicited HAI activity against the strains isolated from 2017-2019 following a single vaccination (**Figure 6A**). Ferrets infected with Pan/99 infected, but only mock vaccinated, had HAI activity against 6/22 (27.27%) of the co-circulating strains, including Nor/10, Nor/11, Mad/11, Ath/12, Den/13, and HK/12/14 (**Figure 6A**).

The ferrets pre-immune to Sich/87 and vaccinated with VLPs expressing TJ-2 HA had HAI activity against 10/22 (45.45%) of the co-circulating strains in the panel, which were all isolated from 2009-2014 with the exception of the Hess/10 and Neth/10 viruses (**Figure 6B**). Animals pre-immune to Sich/87 and vaccinated with VLPs expressing either TJ-5 or Tx/12 HA had HAI activity against the same 13/22 (59.09%) viruses in the panel, which included the strains isolated from 2009-2016, excluding Fiji/16, and Nev/16 (**Figure 6B**). Ferrets pre-immune to Sich/87 and vaccinated with VLPs expressing Wisc/05 HA had HAI activity against 10/22 (45.45%) of the co-circulating strains that were all isolated from 2010-2014 with the exception of the Neth/10 virus (**Figure 6B**). The mock vaccinated Sich/87 pre-immune animals had HAI activity against 5/22 (22.72%) of the co-circulating viruses in the panel, including Nor/10, Mad/11, Ath/12, Den/13, and HK/12/14 (**Figure 6B**).

Animals infected with PC/73 and then vaccinated with VLPs expressing TJ-2 HA had HAI activity against 10/22 (45.45%) of the co-circulating strains, comprising all of the strains from 2010-2014, but excluding Stock/11 (**Figure 6C**). Ferrets pre-immune to PC/73 and vaccinated with VLPs expressing either TJ-5 or Tx/12 HA had had HAI activity against the same 12/22 (54.54%) viruses in the panel, which included all of the strains isolated from 2009-2014 (**Figure 6C**). The ferrets pre-immune to PC/73 and vaccinated with VLPs expressing Wisc/05 HA had HAI activity against 10/22 (45.45%) of the co-circulating strains in the panel, consisting of all the isolates from 2009-2014, but excluding Neth/10 and Stock/11 (**Figure 6C**). The animals that were infected with PC/73 and were only mock vaccinated had HAI activity against 3/22 (13.64%) of the strains that included the Nor/10, Mad/11, and Den/13 viruses (**Figure 6C**). Interestingly the antibodies generated though COBRA vaccination with TJ-5 in animals preimmune to PC/73 were cross-reactive against the co-circulating variants (**Figure 6C**), but not the historical vaccine strains (**Figure 3C**) isolated from 2010-2014.

Overall, the ferrets that were mock infected and then vaccinated with VLPs expressing TJ-2 HA had HAI activity against 4/22 (18.18%) of the viruses in the panel, including the Nor/10, Mad/11, Den/13, and HK/12/14 strains (**Figure 6D**). Ferrets vaccinated with TJ-5 HA had HAI activity against 5/22 (22.72%) of the strains in the panel, including the Nor/10, Mad/11, Ath/12, Jord/12, and Den/13 viruses (**Figure 6D**). Ferrets vaccinated with VLPs expressing either Wisc/05 or Tx/12 HA antigens, or mock vaccinated did not have HAI activity against any of the strains in the panel (**Figure 6D**). Overall, after single vaccination, the pre-immune animals had HAI activity against most of the drift variants isolated from 2009-2014, while the immunologically naïve animals had limited HAI activity against across the panel.

**Figure 7:**
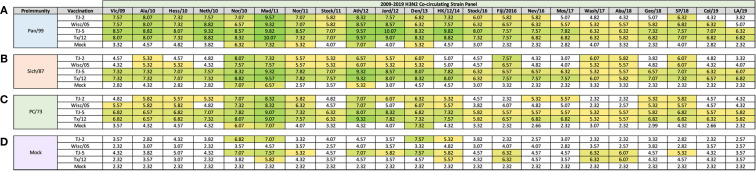
Day 182 H3N2 Co-circulating Strain HAI Panel. Serum collected from animals 182 days post infection (14 days after second vaccination) was analyzed for HAI activity against a panel of co-circulating H3N2 influenza strains spanning 2009-2019. Table is divided into sections based on the virus each animal received to establish preimmunity: A/Panama/2007/1999 **(A)**, A/Sichuan/2/1987 **(B)**, A/Port Chalmers/1/1973 **(C)**, or PBS (Mock) **(D)**. Cells in table are color coded as a heat map based upon Log(2) HAI geometric mean titer (GMT) for each group of ferrets (N=4). The heat map colors cells yellow at Log(2) GMT of 5.32 which correspond to an HAI titer of 1:40 and cells become a darker shade of green as the average antibody titer of the group increases. Cells with no color correspond to groups that did not achieve a GMT ≥5.32.

At 182 days post-infection (14 days post-boost), additional antisera were collected from vaccinated ferrets. In general, after two vaccinations, the Pan/99 pre-immune ferrets had the most cross-reactive HAI activity with the highest magnitude of antibody titers compared to the other pre-immune regimens across the panel (**Figure 7A**). The animals pre-immune to Pan/99 and vaccinated with VLPs expressing either TJ-5 or Tx/12 HA had HAI activity against all 22 strains in the panel from 2009-2019 and those vaccinated with VLPs expressing Wisc/05 HA had HAI activity against all of the strains in the panel, except LA/19 (**Figure 7A**). The TJ-2 HA VLP vaccinated ferrets had slightly less cross-reactive breadth, but still had HAI activity against all of the strains from 2009-2016, as well as the SP/18 strain (**Figure 7A**). The mock vaccinated animals only had HAI activity against 5/22 (22.72%) of the strains in the panel (**Figure 7A**).

Ferrets pre-immune to Sich/87 and then vaccinated twice with VLPs expressing either TJ-5 or Tx/12 HA had HAI activity against all the drift variants from 2009-2019, but at slightly lower titers than the same groups that were made pre-immune to Pan/99 (**Figure 7B**). The groups pre-immune to Sich/87 and vaccinated with VLPs expressing either TJ-2 or Wisc/05 HA had similar patterns of HAI reactivity against the panel of viruses, with HAI activity against 12/22 (54.54%) and 13/22 (59.09%) of the viruses respectively. The Wisc/05 HA VLP vaccines elicited HAI activity against the Hess/10 virus that VLPs expressing TJ-2 HA did not (**Figure 7B**). The mock vaccinated Sich/87 pre-immune animals had HAI activity against 3/22 (13.64%) of the viruses in the panel (**Figure 7B**).

Ferrets that were pre-immune to PC/73, two vaccinations with VLPs expressing either TJ-5 or Tx/12 HA was again sufficient to elicit antibodies with HAI activity against all 22 strains in the panel (**Figure 7C**). However, once again, the overall magnitude of the HAI titer was reduced compared to ferrets that were pre-immune to Pan/99 or Sich/87 (**Figure 7C**). Ferrets pre-immune to PC/73 and vaccinated with VLPs expressing TJ-2 HA had HAI activity against 14/22 (63.64%) of the strains and performed slightly better than ferrets vaccinated with VLPs expressing Wisc/05 HA that had HAI activity to 11/22 (50%) strains (**Figure 7C**). Ferrets pre-immune to PC/73 and then mock vaccinated had HAI activity against 3/22 (13.64%) of the strains in the panel (**Figure 7C**).

Overall, naïve ferrets that were not infected, but vaccinated with VLPs expressing HA vaccines, had antibodies with HAI activity that recognized few viruses in the panel than ferrets pre-immunized with a historical H3N2 viruses (**Figure 7D**). Naive ferrets vaccinated twice with VLPs expressing TJ-2 HA had HAI activity against 4/22 (18.18%) of the viruses in the panel (**Figure 7D**). Naïve ferrets vaccinated with VLPs expressing TJ-5 HA had the most cross-reactivity HAI antibodies compared to other naïve vaccinated ferrets with HAI activity against 11/22 (50%) of the drift variants in the panel (**Figure 7D**). Vaccination of naïve ferrets with VLPs expressing the Tx/12 HA had HAI activity against 5/22 (22.72%) of the viruses, while VLPs expressing the Wisc/05 HA and mock vaccinations did not generate seroprotective HAI antibody titers against any of the strains in the panel (**Figure 7D**). In naïve animals, the COBRA HA VLP vaccinations induced more cross-reactive antibody breadth than the era matched WT HA VLP comparators.

### H3N2 Neutralization Assays

**Figure 8:**
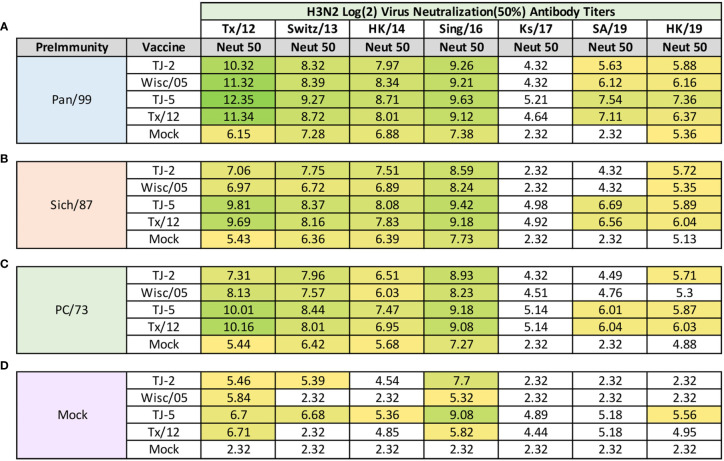
Day 98 H3N2 Focal Reduction Assays. Serum collected from animals 98 days post infection (14 days after first vaccination) was analyzed for neutralizing antibody activity against a panel of historical H3N2 influenza vaccine strains spanning 2012-2019 using a focal reduction assay. Table is divided into sections based on the virus each animal received to establish preimmunity: A/Panama/2007/1999 **(A)**, A/Sichuan/2/1987 **(B)**, A/Port Chalmers/1/1973 **(C)**, or PBS (Mock) **(D)**. Cells in table are color coded as a heat map based upon Log(2) HAI geometric mean titer (GMT) where 50% virus neutralization (Neut50) was observed for each group of ferrets (N=4). The heat map colors cells yellow at Log(2) GMT of 5.32 which correspond to an antibody titer of 1:40 and cells become a darker shade of green as the average antibody titer of the group increases. Cells with no color correspond to groups that did not achieve a Neut50 GMT ≥5.32. The lowest serum dilution analyzed was 1:20, if no Neut50 titer was observed at this dilution a Neut50 GMT value of 2.32 which corresponds to an antibody titer of 1:5 was assigned to the group.

Influenza focal reduction assays (FRAs) were used to determine the ability of vaccine elicited antibodies to neutralize live virus infections against a panel of 7 historical influenza A(H3N2) vaccine strain isolates from 2012-2019 at day 98 post infection (**Figure 8**). All of the ferrets pre-immune to Pan/99 and vaccinated one time, regardless of vaccine, had Log_2_ 50% neutralization (Neut_50_) titers that were superior to mock vaccinated animals across all of the viruses in the panel (**Figure 8A**). In general, for the Pan/99 pre-immune animals, TJ-5 produced the highest magnitude Neut_50_ titers against every virus in the panel compared to the other vaccine antigens (**Figure 8A**). The lowest Neut_50_ titers from this pre-immune regimen were produced against the Ks/17 virus, against which none of the vaccines produced a Neut_50_ titer greater than 1:40 (**Figure 8A**). An antibody titer of 1:40 in this assay does not correlate with a sero-protective titer, but it is a correlate for sero-protection for the HAI assay, so we used this as a baseline to make comparisons between responses.

Ferrets pre-immune to Sich/87 and vaccinated with VLP antigens also consistently produced higher Neut_50_ titers than those that received mock vaccine (**Figure 8B**). Again, TJ-5 produced higher Neut_50_ titers across the panel of H3N2 viruses than any other vaccine, with the exception of Tx/12 against HK/19 where it slightly outperformed TJ-5 with a Log_2_ Neut_50_ titer of 6.04 compared to 5.89 (**Figure 8B**). Much like the Pan/99 groups, the Sich/87 pre-immune groups also had trouble generating neutralizing antibody titers greater than 1:40 against the Ks/17 virus, and additionally against the SA/19 virus where only TJ-5 and Tx/12 vaccines generated Neut_50_ titers greater than 1:40 (**Figure 8B**).

Much like the Pan/99 and Sich87 pre-immune ferrets the PC/73 pre-immune animals vaccinated with any of the H3 VLP antigens generated a strong neutralizing antibody response against the viruses from 2012-2016 (**Figure 8C**). The TJ-5 and Tx/12 antigens generated the highest antibody responses across the panel, and those antigens also possessed Neut_50_ titers greater than 1:40 against both SA/19 and HK/19 (**Figure 8C**). None of the vaccine antigens produced Neut_50_ titers greater than 1:40 against the Ks/17 isolate in the PC/73 pre-immune animals (**Figure 8C**). All four of the vaccines induced high neutralization titers against the Tx/12 and Sing/16 viruses in the influenza naïve mock pre-immune groups (**Figure 8D**). In this group TJ-5 induced the most breadth and produced the highest neutralization antibody titers across the panel of viruses, with its highest response being against the Sing/16 virus (**Figure 8D**). Mock pre-immune animals vaccinated with TJ-2 produced Neut_50_ titers greater than 1:40 against 3 of the viruses in the panel while Tx/12 and Wisc/05 only achieved this feat against 2 of the viruses, Tx/12 and Sing/16 (**Figure 8D**).

## Discussion

OAS to influenza viruses is a phenomenon that was first recognized in the 1960’s and has been observed in many different clinical studies and animal models under controlled conditions (50). However, recapitulating the human immune response in relevant influenza virus animal models, like the ferret, is difficult. Multiple studies have demonstrated that ferrets and humans have different antibody responses to influenza virus infection, primarily because humans have extensive immune histories with the virus through natural infections and vaccinations (17). The influenza virus strains that a person encounters within the first few years of life can leave lasting impacts that effect how an individual responds to antigenically distinct viral strains later in life (51, 52). The study presented herein aimed to investigate the effects of historical A(H3N2) influenza virus imprinting on the elicitation of antibodies that recognize a broad number H3N2 viruses following subsequent vaccination with VLP vaccines expressing either COBRA or wild-type H3 HA antigens. Ferrets were initially primed using live A(H3N2) influenza virus infection using representative viruses isolated from the 1970’s, 1980’s, and 1990’s to model a person’s first infection with the virus during these eras. VLPs expressing the COBRA or WT HA vaccine antigens were then evaluated in this animal model to determine their elicitation of antibodies against drifted strains of influenza virus, since ideal vaccine candidates should induce robust immune responses in all individuals, regardless of their specific immune history.

Differences in COBRA and WT HA VLP vaccine performances were observed between groups of ferrets depending on their specific immune histories to A(H3N2) influenza viruses. In general, VLPs expressing H3 HA vaccine antigens that were more genetically similar to the specific priming virus induced the highest level of HAI breadth across the panel of historical A(H3N2) influenza vaccine strains (**Figure 3**). VLPs expressing the TJ-5 and Tx/12 HA antigens induced the most breadth of HAI responses in ferrets primed with the Pan/99 or Sich/87 viruses. The HA proteins of these vaccines share ~91% genetic similarity to both of the priming strains (Pan/99 and Sich/87). After one vaccination, these vaccines elicited seroconversion to 56-80% viruses in the panel. However, ferrets pre-immune to the PC/73 virus and then administered these same vaccines elicited antibodies with HAI activity against only 16-20% of the viruses in the panel. The PC/73 HA is ~87% similar to the two vaccine strains. In this study it appears that when the genetic distance between priming strain and vaccine antigen is greater ~87%, the ability of the HA vaccine antigens to elicit broadly-reactive antibodies against the panel drops significantly. This is potentially a result of the number of shared antigenic epitopes between the priming strain and the vaccine antigen. Genetic similarity between the two could possibly present the immune system with more shared antigenic epitopes that can be recalled by vaccination, leading to a more robust antibody response (10).

However, in this study it appears that this genetic distance bias can be overcome through repeated vaccination with the same H3 antigen. After boosting the PC/73 primed ferrets with a homologous vaccination of VLPs expressing TJ-5 or Tx/12 HA, the seroconversion rates across the panel of historical vaccine isolates rose from 16-20% to 60-72% (**Figure 4**). A similar phenomenon also occurred in the 2009-2019 co-circulating A(H3N2) strains panel (**Figure 6**). After one vaccination with VLPs expressing either TJ-5 or Tx/12 HA, the ferrets seroconverted to 45-59% of the strains regardless of the priming virus, but none of the animals had seroprotective titers against any of the strains isolated from 2017-2019 (**Figure 6**). Although seroconversion rates against this panel were similar amongst all of the pre-immune ferrets, ferrets primed with the genetically distant PC/73 virus had a drop in HAI titers compared to the ferret’s pre-immune to either Sich/87 or Pan/99. After the second vaccination VLPs expressing the TJ-5 or Tx/12 HA, all of the pre-immune ferrets seroconverted to all of the strains in the panel, regardless of the priming virus (**Figure 7**). This was in stark contrast to the immunologically naïve animals that seroconverted to at most 50% of the co-circulating A(H3N2) strains after two vaccinations with VLPs expressing TJ-5 HA. Overall, it appears that the H3 vaccine candidates induced more seroconversion in animals that possess an immunological history with A(H3N2) influenza strains and the differences in immunological reactivity driven by that history can be overcome with repeated vaccination. This is likely due to the generation of new memory B cell populations elicited by the first vaccination, which can then be recalled upon subsequent vaccination with the same antigen (53). However, current limitations in ferret B cell immunology and the availability of ferret immunoassay reagents prevent us from investigating these questions within our current study, therefore future experiments will be aimed at answering these types of questions.

All of the A(H3N2) pre-immune ferrets that received vaccines, COBRA or WT HA, also had a high level of neutralizing antibody activity across the panel of A(H3N2) historical vaccine viruses from 2012-2019, with the exception of the Ks/17 virus (**Figure 8**). The Ks/17 virus is slightly different from most of the other viruses in the panel as it, as well as Switz/13 belong clade 3c3.a. Other viruses in the panel either belong to clade 3c2 or its subclades 3c2.a and 3c2.a1. 3c3.a viruses elicit antibody responses in ferrets that are biased toward antigenic site B (17). 3c3.a viruses possess a K160 in site B, while 3c.2a viruses possess a T160 that results in the addition of an N-linked glycosylation at site 158 (17). This glycosylation present on the HA of 3c2.a viruses may cover up antigenic epitopes that are necessary to generate strong neutralizing antibody responses to 3c3.a viruses (51). Switz/13 and Ks/17 differ by 4 amino acids (AA) in antigenic site B, N160K, G202V, D206N, and F209S, which may also drive the observed differences in their neutralization titers, but neither possess the glycosylation motif at site 158 that is present in the 3c2.a viruses. The neutralization assays performed in this study also take into account the contributions of non-HA reactive antibodies, such as antibodies directed against the viral NA, obtained from the virus priming, that may play a role in the observed differences in neutralization titers across the panel. This may play a significant role as animals that did not possess immunologic memory to the NA of the A(H3N2) priming viruses did not acquire high neutralization titers after repeated vaccinations with H3N3 VLP vaccines. Additionally, differences were observed in the antibody reactivity between the HAI and FRA assays. The HAI assay predominately detects antibodies directed towards the head region of the HA molecule that prevent binding of the HA with the host sialic acid. Antibodies directed towards the stem of the HA protein typically will not show up in this analysis as they can still allow this interaction between the HA head and the host sialic acid to take place. These antibodies still possess the ability to prevent viral replication, but typically this is done by preventing the conformational changes that take place in the endosome once the virus has entered into the host cell preventing membrane fusion or cleavage of the HA0 into HA1 and HA2 fragments (54). In contrast to the HAI, the FRA detects all antibodies that possess the ability to prevent viral infection. It is likely that the differences in HAI and FRA titer are being driven by HA stem reactive antibodies that do not play a role in HAI based protection. However, HAI specific antibodies are typically immunodominant over stalk-specific antibodies, and also tend to generate more effective neutralizing immune responses, lower IC50; therefore, antigen design methodologies such as COBRA which drive HA head-based antibody responses could be highly advantageous for generating potent, cross-reactive immune responses (12, 55–57).

The ferrets vaccinated with antigens representing the early 2010’s, VLPs expressing TJ-5 and Tx/12 HA, possessed the most cross-reactive HAI antibodies in all three pre-immune regimens, centered around A(H3N2) viruses isolated from 2002-2019. These two antigens are quite similar, as they differ by only 9AA (1 AA in antigenic site A, 2 in site B, and none in the other antigenic sites C-E). Both possess 12 potential glycosylation site motifs, including the glycosylation motif at site 158, generate similar patterns of HAI cross-reactivity, and display similar live virus neutralizing ability across the panels. The similar HAI antibody profiles elicited by these two vaccines could in part be attributed to the cross-reactivity of the A(H3N2) vaccine strains isolated from 2009-2016 (33). The majority of these viral isolates belong to clade 3c2 or its subclades 3c2.a and 3c2.a1 that tend to elicit HAI cross-reactive antibodies to one another (27). What differentiates these two vaccines are the magnitude of antibody titer that is generated after one vaccination in the pre-immune model. In most cases, for both the Pan/99 and Sich/87 pre-immune ferrets, TJ-5 HA consistently produces higher HAI antibody titers than Tx/12 HA across the panel after just one vaccination (**Figure 3**); while VLPs expressing Tx/12 HA appear to require 2 vaccinations to achieve antibody titers that are similar to those elicited by VLPs expressing TJ-5 HA (**Figure 4**). A similar trend was observed with the two vaccine candidates representing the 2002-2005 era, TJ-2 and Wisc/05 HA proteins. These are also similar antigens to one another as they differ by only 8AA (2 differences in antigenic site A, 2 in site B, and none in the other antigenic sites C-E). Much like VLPs expressing TJ-5 HA, TJ-2 HA was more cross-reactive in the HAI assay, in the pre-immune ferrets, than Wisc/05 HA after one vaccination (**Figure 3**). After 2 vaccinations with VLPs expressing Wisc/05 or TJ-2 HA, antibody titers across the historical vaccine strain panel increased to similar levels for both vaccine antigens (**Figure 4**). The HAI reaction for TJ-2 does decrease against 2 of the viruses from day 98 to day 182. This decrease is not great in magnitude, but it is enough to cause the animals to fall below the 1:40 seroconversion cutoff. This drop in titer is likely the result of B cells undergoing affinity maturation toward epitopes present in the vaccine antigen, and making the animal’s immune responses more tailored to targeting vaccine-based epitopes rather than ones present on the Sich/87 priming strain. After one vaccination the animals possess memory cells to both the priming strain and vaccine strain that can be preferentially recalled by the second vaccination. The number of strains that the vaccinated animals seroconvert to also increases from day 98 to 182, and at the later timepoint future drifted strains from 2016-2018 that were not seroconverted against after one vaccination are now being picked up. This phenomenon could also be explained by B cell affinity maturation; whereby epitopes present on the vaccine that are not shared with the Sich/87 priming strain are driving the immune response towards recalling new epitopes that are shared between the vaccine and future drifted strains that may not be present on past viruses.

In this study, it appears that the VLPs expressing COBRA HA vaccine antigens are superior at eliciting cross-reactive HAI antibody breadth after one vaccination than VLPs expressing the WT HA vaccine antigens. Additionally, VLPs expressing the WT HA vaccines require a boost to elicit similar levels of breadth as VLPs expressing the COBRA HA antigens. This is particularly evident in the Sich/87 pre-immune groups (**Figure 3B**). Animals vaccinated with TJ-2 induced seroconversion against 7/25 strains in the panel, while the WT comparator, Wisc/05, only induced seroconversion against 4/25 strains. Furthermore, animals vaccinated with TJ-5 induced seroconversion against 20/25 strains in the panel, while its WT comparator Tx/12 only induced seroconversion against 12/25 strains. The COBRA vaccines also do as good, or better than the WT comparators in the Pan/99 pre-immune group after one vaccination. TJ-2 induces seroconversion to the same number of strains as Wisc/05, and TJ-5 induces seroconversion to 3 more strains than Tx/12. Additionally, TJ-5 induces higher magnitude HAI titers than Tx/12 (**Figure 3A**), in 11 of the 14 viruses that they both induce seroconversion against. These cross-reactive responses and high magnitude antibody titers generated by the COBRA antigens after just one vaccination are highly advantageous, as most commercial influenza vaccines are delivered as single dose vaccines; whereas the WT vaccine antigens used as comparators in this study required two doses in order to achieve a similar immune response to those elicited by the COBRA vaccines.

In some cases, WT antigens such as Tx/12 can produce more cross-reactive antibodies than other WT strains, and this is evidenced by recent seasons where H3 WT vaccine efficacy is much lower in humans than in the previous season (14, 17, 18). For comparison, the Wisc/05 vaccine in this study is much less cross-reactive than Tx/12 in all 3 pre-immune settings (**Figure 3**). This phenomenon is likely dependent on the number of shared antigenic epitopes between the chosen vaccine strain and those present in circulating viruses. The COBRA methodology aims to decrease the chances of having a mismatched vaccine strain by producing an optimized antigen that contains multiple antigenic epitopes from a number of different recently circulating viruses, in order to maximize the cross-reactive potential of the induced antibody pool.

Similar to other broadly reactive influenza vaccine candidates, COBRA HA vaccines likely generate B cell responses that target conserved epitopes on the HA molecule that are present on both historical and modern isolates of A(H3N2) influenza (25). Studies utilizing monoclonal antibodies generated from COBRA HA antigens have shown a preference for binding conserved epitopes on the HA globular head, such as the receptor binding site, while others are directed at the stem region of COBRA HA (55). This is advantageous, as antibodies that bind conserved regions of the HA protein can provide protection against multiple influenza viruses (56). Additionally, antibodies directed against the HA globular head induce higher titers and have. more neutralizing potency than those directed against the HA stalk region (12, 56, 57). However, it is still unknown whether the cross-reactivity of COBRA HA generated antibody responses are the result of polyclonal antibodies working together to target multiple conserved epitopes or specific monoclonal responses targeting a single epitope (55). Future studies will focus on determining the COBRA HA mechanisms of action that elicit broadly reactive antibodies, as this will provide valuable information on improved vaccine design.

There are some limitations to the pre-immune ferret model presented in this study. First, this model only used one influenza A(H3N2) infection to establish pre-immunity. Most people have a much more extensive immune history from influenza virus infections, stemming from multiple infections and vaccinations with multiple subtypes of influenza throughout the course of their lives (9). Using a more diverse regimen of infections and vaccinations that include A(H1N1) and influenza B antigens to establish pre-immunity in ferrets may represent the immune state of an adult human more accurately. Additionally, all of the ferrets used in this study were the same gender and approximately the same age. Influenza vaccine effectiveness is known to vary depending on vaccination history, age, sex, and high-risk condition (53). Differences in immunological memory and responses to vaccination may exist between genders and age groups and should be investigated in future studies. Also, this model is set up to represent individuals who were first exposed to A(H3N2) influenza during the early part of their lives, but it does not accurately depict the differential immune state of individuals born in the 1970’s, 1980’s and 1990’s. Older individuals first exposed in the 1970’s should have more extensive immune histories with influenza than an individual born in the 1980’s and 1990’s (24). Also, the effects of immunosenescence that may be present in older individuals are not accounted for in this study, as all of the ferrets were the same age at the time of infection and vaccination (58). The use of VLP vaccines in this study may also generate different immune responses in pre-immune animals than more conventional vaccine antigens like live attenuated (LAIV), split inactivated (IIV), or subunit influenza vaccines. Live influenza virus vaccines, for example, induce superior immune responses because they are administered *via* the natural route of infection, intranasally, and drive diverse adaptive immune responses including secretory IgA, serum IgG, and cell mediated responses; while inactivated vaccines tend to primarily induce a serum anti-HA IgG antibody response (9, 24).

Future studies will be aimed at investigating the effects of pre-immunity with heterologous subtypes of influenza viruses on vaccination with monovalent formulations or cocktails of universal H1 and H3 COBRA HA vaccine candidates. It is currently unknown how universal vaccine candidates will perform in a population that has a diverse pre-existing immune history to A(H1N1), A(H3N2), and influenza B subtypes. The impact of whether an individual’s first exposure to an A(H1N1) or A(H3N2) influenza virus can have a large influence on future immune responses (52, 59). Imprinting with one subtype of influenza may make it more difficult to generate antibodies to other heterologous subtypes later in life (24, 52). Additionally, most influenza virus vaccines are administered as cocktails containing antigens from multiple subtypes of influenza A, H1N1 and H3N2, and influenza B (60, 61). Therefore, priming with one subtype of influenza may bias an individual to generating antibodies against that particular subtype upon subsequent vaccination, as immunodominance of one subtype over another may also play a role in the elicited immune responses. This is an important aspect to understand, since antigens from HA group 1 (*e.g*. H1, H5) appear to induce narrower immune responses and less cross-group protection than HA group 2 antigens (*e.g.* H3, H7) (52). Studies designed to more accurately recapitulate the complex immune histories of humans are greatly needed and will provide valuable information that can be used to design universal vaccine candidates, such that they are effective in all populations, regardless of an individual’s unique pre-existing immune history to influenza virus.

## Data Availability Statement

The original contributions presented in the study are included in the article/supplementary material. Further inquiries can be directed to the corresponding author.

## Ethics Statement

The animal study was reviewed and approved by University of Georgia IACUC.

## Author Contributions

JA and TR conceptualized the experiments. JA designed and prepared the vaccines, conducted the animal work, collected samples, performed serological assays, and prepared the figures. JA analyzed the data and wrote the manuscript with input from TR. All authors contributed to the article and approved the submitted version.

## Conflict of Interest

The authors declare that the research was conducted in the absence of any commercial or financial relationships that could be construed as a potential conflict of interest.

## Publisher’s Note

All claims expressed in this article are solely those of the authors and do not necessarily represent those of their affiliated organizations, or those of the publisher, the editors and the reviewers. Any product that may be evaluated in this article, or claim that may be made by its manufacturer, is not guaranteed or endorsed by the publisher.
